# Case of a Compound Dislocation of the Tibia and Fibula, Accompanied with a Fracture and Loss of a Considerable Portion of the Astragalus, and Likewise with a Fracture of the Thigh Bone; with Remarks

**Published:** 1794

**Authors:** James Rumsey

**Affiliations:** Surgeon at Amersham in Buckinghamshire.


IV. *Case of a compound Dislocation of the Tibia and Fibula, accompanied with a Fracture and Loss of a considerable Portion of the Astragalus, and likewise with a Fracture of the Thigh Bone; with Remarks. By Mr.* James Rumsey, *Surgeon at Amersham in Buckinghamshire.*

ON the 21st of June, 1792, Mr. Tolson, aged forty years, a reputable tradesman in New Bond Street, Westminster, was thrown from a curricle on Gerrard's-Cross Common, eight miles from this place, in consequence of the horses taking fright, and drawing the carriage with great velocity against a tree. The injury he received from this accident consisted in a compound dislocation of the tibia and fibula at the outer ancle of the left leg, with a fracture of the astragalus, (the superior half of which was attached to the dislocated bones of the leg) and likewise (though, as we shall see, not immediately noticed) a simple fracture of the os femoris on the same side. He was immediately conveyed to a friend's house on the common, where he had the advantage of an airy, healthy situation, with every kind do mestic attention the family could administer.

I saw

I ſaw him about two hours after the accident, and found the bones protruding at the ancle through a very large wound, with the foot turned inwards and upwards; and the integuments, beneath the wound, exceedingly confined by the diſlocated bones, which deſcended nearly to the bottom of the foot. A conſiderable hæmorrhage had taken place, but was ſtopped by the ſpontaneous contraction of the lacerated veſſels.

From ſuch a formidable accident in ſo large a joint, there appeared very little probability of the patient's recovery, without immediate amputation. I therefore requeſted that a conſultation with ſome other ſurgeons might be expeditiouſly held on the caſe, and expreſſes for this purpoſe were accordingly ſent to Mr. Pearſon, ſurgeon in London; and to my brother, Mr. Henry Rumſey, ſurgeon at Cheſham, in this county. While I was waiting for their arrival, the patient requeſted me to examine his thigh, which till then he had not particularly noticed, when I plainly diſcovered an oblique fracture of the os femoris, at its ſuperior part. This additional evil appeared to me a great obſtacle to an amputation.

My brother, when he arrived, being of a ſimilar opinion, I attempted to reduce the fractured diſlocated joint into its proper ſituation. This

This I found very difficult to effect, without firft feparating that part of the aftragalus which was pendulous to the tibia, having its capfular ligament lacerated one half way round the joint *; I therefore removed it without hefitation, being perfuaded, that if it had been practicable to reduce it into its original fituation, fo large and moveable a portion of bone would have been a fource of pain and irritation, and have rendered the cure more difficult and uncertain.

I then divided that portion of the integuments of the foot which was confined by the protruded end of the tibia, which enabled me with eafe to reduce it and the fibula into their proper fituation. I applied fome doffils of lint, dipped in tincture of opium, to the wound, and covered the whole with a poultice of ftale beer and oatmeal. We now reduced the fractured fe-

* This portion of the aftragalus confifts of the broad fmooth head by which it is articulated to the tibia ; of almoft the whole of the inner and outer fides of this head by which it moves on the inner and outer malleoli ; and of about the upper half of the pofterior cavity on its under furface, by which it is united to the os calcis ; fo that the bone was divided nearly horizontally, and the part left behind confifts of the lower half of the laft-mentioned cavity, and of the whole of the other or anterior cavity which connects it with the os calcis ; and of the anterior portion or procefs by which it is articulated to the os naviculare.

mur, mur, and placed the limb in the bent pofition, expecting that our greateft fuccefs would be in procuring a complete anchylofis, the failure of which, I concluded, would leave a ufelefs foot. The under fplinter was a firm excavated piece of deal, of the fhape of the leg and foot, with a hole oppofite the ancle.

Mr. Pearfon arrived in the evening, and approved of the preceding treatment, giving it as his opinion that it would be fafer to attempt the prefervation of the limb, than to amputate under fuch complicated circumftances. The wound was concealed as much as poffible from the external air, and the cataplafm renewed no oftener than the difcharge rendered neceffary.

22. The preceding night had been very painful, with delirium and vomitings; the pulfe was full and frequent; I took away ten ounces of blood, and gave Kali Tartarifat. and Manna, in dofes fufficient to procure ftools. A common faline draught, with antimonial wine and tincture of opium, was given every four hours, and a fuller dofe of tincture of opium at bed-time.

23. The vomiting continued; the ancle and thigh had been lefs painful through the night; the faline draughts were continued, but without the antimonial, on account of the vomiting. During this period the antiphlogiftic regimen was ftrictly adhered to.

24. The night had been tolerable; the vomiting had ceafed; the pulfe was fofter; the faline draughts were continued, with the opiate, at bed-time. This evening the leg was very painful.

25. He had had a pretty good night; a difcharge from the wound now commenced, and the tenfion of the mufcles of the thigh began to diminifh.

26 and 27. The fame treatment was continued. The difcharge increafed, and the tenfion of the thigh was much abated.

28. The ancle was much fwelled and inflamed; I therefore exchanged the beer grounds in the cataplafm for the *Aq. Litharg. Acetat.* The patient had this day much pain in the bowels from flatulence; from which circumftance, and that of the difcharge being very thin, it was judged expedient to vary his mode of living, and likewife his medicines.

29. He was allowed a fmall portion of animal food, fome table beer, and fome port wine; and he took the bark liberally, both in fubftance and decoction. This change of treatment agreed with him perfectly well. At this time I found it neceffary neceſſary to alter the poſition of the limb, on account of the preſſure on the wound, occaſioned by its laying in the bent poſition, and the pain it gave in turning to dreſs it, which, from the copious diſcharge, there was now a neceſſity of doing night and morning. I therefore placed it on the heel, uſing the common deal flexible ſplint, of the length of the limb, and confined it in a box, whoſe ſides and lower end let down. The ſpace between the ſides of the box and the ſplint was filled with pieces of flannel. By theſe means, and the uſe of the eighteen-tail bandage, the dreſſings were applied with very little diſturbance to the leg, whereby the patient eſcaped much pain: the upper end of the box under the ham was raiſed; which gave the muſcles ſome degree of flexion, and, at the ſame time, was favourable to the diſcharge.

The foot having a tendency to fall inwards, and the end of the fibula to protrude through the wound, it required great attention to prevent the deformity the neglect of theſe circumſtances might have occaſioned. The mode of prevention I adopted, and which proved ſucceſsful, conſiſted in employing a number of ſmall deal wedges, about ſix inches long, two broad, and a quarter of an inch thick; as many of theſe as

 were were found ſufficient were placed oppoſite the inſide of the foot, between it and the ſide of the box; others in the ſame manner on the outſide of the calf of the leg; by which means the limb was kept ſteady: and by placing the heel eaſy, and rather hollow, none of the uſual evils ariſing from preſſure on the heel occurred.

30, The bark agreed very well; the opiate was continued at bed-time; the diſcharge was great, but more purulent; the pulſe was become ſofter and leſs frequent; and the urine, which hitherto had been clear and very high coloured, was now turbid: the pain and inflammation being much diminiſhed, the cataplaſm was diſcontinued, and the wound dreſſed with dry lint, with a pledget of *Cerat. Litharg. acet. c.* over it, and a moderate compreſſion was made by means of the bandage. From this period the wound progreſſively mended; the diſcharge diminiſhed; granulations formed; and the ſurrounding ſkin began to heal. The uſe of the bark and of the opiate was continued till the beginning of Auguſt. About the end of July the progreſs of the cure was retarded by matter, collected under the integuments above the inner ancle, which, on preſſure, came out at the wound. After trying the effects of permanent nent preſſure, for the prevention of this depoſit, in vain, I made an inciſion into the cavity, and filled it with dry lint to produce inflammation on its internal ſurface, which perfectly conſolidated it; and from this time the wound rapidly healed, and became perfectly cicatriſed by the middle of September, without any exfoliation of bone, larger than the head of a pin, having taken place.

The fracture of the femur went on very well, excepting that the obliquity of it, with the impoſſibility of producing a permanent extenſion, on account of the leg, occaſioned a degree of curvature, which it otherwiſe would not have had.

The limb gradually acquired ſtrength; and it is a very remarkable fact, that the patient is now able to walk very well, with only the aid of a ſmall ſtick, and even this aſſiſtance he will probably not require long. There is no anchyloſis to render the ancle immovable; but a ſufficient firmneſs has been produced in the ſurrounding parts, by the long-continued inflammation, to aſſiſt in the formation of an artificial joint, which poſſeſſes a degree of motion nearly equal to that of the natural one.

The refult of this cafe affords an additional argument againft immediate amputation in cafes of bad compound fracture; a fubject on which furgeons, eminent in their profeffion, have been much divided.

The impure air of London and other great cities, but more particularly of large hofpitals in fuch fituations, probably renders thefe accidents more generally fatal there than in the country; and the conftitution of many patients is, no doubt, more endangered in cities, by the long-continued copious fuppuration confequent to extenfive injuries, than it would be by amputation; and cities being commonly the refidence of the moft eminent men, thefe circumftances may have occafioned fome of them to lay it down as generally neceffary to amputate in cafes of bad compound fracture: but in the country I fhould ever attempt to fave the limb, except in cafes where the divifion of large blood veffels, or a confiderable lofs of the mufcles, fhould call for immediate amputation. I believe that many of thofe cafes which prove fatal without the operation, would have a fimilar termination if it were performed, and *vice verfa*. The caufe of their fatality feems oftentimes to depend on fome-

ſomething unfavourable in the habit of body; and this renders it neceſſary for the ſurgeon to diſcover, as clearly as he can, the preceding ſtate of his patient's health, and very ſtudiouſly to watch the progreſs of each day in the early part of all formidable accidents, as this only can direct him in what degree he is to adopt, and how far continue, the relaxant, antiſeptic, or tonic modes of treatment; as in caſes of this kind, no one general rule of conduct will be equally ſucceſsful.

The ſame attention will be neceſſary to be obſerved reſpecting the local treatment, that irritation and pain may be avoided as much as poſſible.

The poſition of the limb in compound fracture muſt depend on the ſituation of the wound. In ſimple fracture, during the proceſs of ſwelling and inflammation, great advantage reſults from the bent poſition, which is certainly leſs painful, from the muſcles being relaxed; but when the tenſion has entirely ſubſided, I confeſs I keep the fractured ends of the bones better in their place by the ſtraight poſition; and the time of confinement being divided in the two ſituations, the patients generally bear it better; and when the union is perfect, flexion may be again permitted with propriety.

*Amersham,*

*March* 31, 1793.

